# Current Imaging Trends in COVID-19 Pneumonia

**DOI:** 10.31729/jnma.4992

**Published:** 2020-06

**Authors:** Anamika Jha, Benu Lohani, Ram Kumar Ghimire

**Affiliations:** 1 Department of Radiology, Tribhuvan University Teaching Hospital, Maharajgunj, Kathmandu, Nepal; 2 Department of Radiology, Nepal Mediciti Hospital, Bhaisepati, Lalitpur, Nepal

**Keywords:** *COVID-19 pneumonia*, *chest X-ray*, *CT scan*

## Abstract

COVID-19 has rapidly emerged as a pandemic threatening lives and healthcare systems worldwide. With the emergence of the disease in Nepal, all faculties of medicine need to be well prepared to face the challenge. Fortunately, now plenty of research is available to facilitate our preparedness in the war against COVID-19. The reverse transcriptase-polymerase chain reaction is the current gold standard diagnostic test and chest Computed Tomography scan for screening the disease is considered inappropriate by most society recommendations. The Nepal Radiologists’ Association has proposed its guidelines which have been endorsed by the Nepal Medical Council. This article aims to summarize the role of imaging focusing on chest X-ray and Computed Tomography scan including the indications, specific findings, and important differentials. Imaging needs to be done taking necessary precautions, to minimize disease transmission, protect health care personnel, and preserve health care system functioning.

## INTRODUCTION

COVID-19 cases are increasing in Nepal. This disease has a high transmission rate and primarily spreads via respiratory droplets or fomites. There is an urgent need for medical professionals to be prepared to face the challenges posed by this disease so that the disease can be contained, diagnosed early, and treated. Reverse transcriptase-polymerase chain reaction (RT-PCR) is the current gold standard diagnostic test, though limited by its low sensitivity and longer turnaround times.^1^ To facilitate radiological services in Nepal, ensuring the health of the involved professionals, and obtaining maximum infection control, the Nepal Radiologists’ Association has also proposed its interim guidelines.^2^

In this article, we summarize the role of imaging in COVID-19 pneumonia, highlighting that necessary precautions must be taken to minimize disease transmission to protect health care personnel and preserve health care system functioning.

**IMAGING MODALITIE**S

The primary imaging modalities involved in the diagnosis of COVID-19 pneumonia are chest X-ray and high-resolution CT scan. There is a role of CT pulmonary angiography in patients with complications where pulmonary embolism is suspected. The role of point of care ultrasound (POCUS) is not discussed in most of the guidelines. While POCUS may be a reasonably cost-effective way for monitoring disease at the bedside, it is limited by several factors like expertise, availability of portable ultrasound machines, decontamination of the system to ensure infection control, and need for another modality with a positive POCUS study. Additionally, POCUS may diagnose pleural or peripheral lung lesions, but will completely miss it if there is a normal lung between the lesion and pleura.

**INDICATIONS FOR IMAGIN**G

There is no role of CXR or CT scan for screening or in patients with mild disease. Imaging CXR or CT scan is needed in COVID patients with worsening symptoms and recovered patients with persistent hypoxemia or respiratory impairment or both.^3^ CT angiography may be indicated in patients with worsening symptoms and to rule out complications like pulmonary embolism. In a patient with moderate to severe symptoms with negative RT-PCR, HRCT chest may be helpful, and with the presence of typical imaging features, the diagnosis of COVID-19 may be made, repeating RT-PCR for confirmation at a later date. Fleischner society has not recommended daily CXR in intubated but otherwise, stable patients.^4^

CT scan is more sensitive and specific than CXR and can also predict the prognosis, i.e. those with more lungs involvement may be far worse clinically, compared to those with no or minimal changes. Few studies have suggested chest CT scans for screening, comprehensive evaluation, and follow up in epidemic areas highlighting its high sensitivity for early diagnosis of COVID-19 compared to RT-PCR.^5^ However, these studies though contributory to our knowledge, are mostly retrospective reviews of low quality and considered level 3 body of evidence.^6^ The use of CT scan would depend upon the medical infrastructure, availability of CT scanners, medical manpower, effective deep cleaning and infection control measures at the workplace, the absence of any of which, would not only challenge the role of CT scan but also promote the spread of infection.

Choosing the use of appropriate modality, that is, CXR or CT scan depends upon its availability, mobility of the patient, and ease of deep cleaning. A dedicated portable modality like X-ray machine will help in diagnosis, especially in the difficult to mobilize patients and prevent contamination of places which may be more time taking and difficult to decontaminate.

**IMAGING FEATURE**S

CXR may be normal in the first 4-5 days of symptom onset while CT scan may show abnormalities earlier. Imaging findings are maximum at 10-12 days.^7^ Most frequent findings on CXR, which has a sensitivity of 69% compared to 91% for RT-PCR test, include bilateral, peripheral lower zone consolidation.^8,9^

Findings on CT scan include ground-glass opacity (GGO) with round morphology or crazy-paving, reverse halo sign, consolidation in a peripheral, posterior, and diffuse or lower lung zone distribution. Pleural effusion and lymphadenopathy are unusual when alternative diagnoses should be considered. With temporal evolution complete resolution, multifocal organizing pneumonia and architectural distortion in a peripheral distribution may be seen.^10^ For uniformity and effective communication standardized reporting format has been suggested which categorize findings as typical, probable, or indefinite for the diagnosis.^11^ Lexicons have also been developed, like CO-RADS and COVI-RADS for COVID-19 Reporting and Data System, for describing the imaging findings and grading the severity of infection for prognostication.^12^,^13^

**DIFFERENTIAL DIAGNOSI**S

The imaging features may overlap with other infections like influenza. Causes of ground glass haziness or consolidation like Pneumocystis jerovici pneumonia, non-specific interstitial pneumonia, and pulmonary edema are other differentials.^14^ These, however, have a different distribution in lung parenchyma with central distribution and subpleural sparing.^15^ Pulmonary edema which may be seen in the COVID patients as well as developing secondary to heart failure precipitated by fever and dehydration or myocarditis, has central ground glass density, interlobular septal thickening with pleural effusion. Tuberculosis is often seen in our subcontinent and when it is co-existent COVID pneumonia, may be difficult to differentiate, however in the majority of cases it has upper lobe and apical-basal segment involvement. Bacterial pneumonia including tuberculosis often has pleural collections and lymphadenopathy.

COVID-19 pneumonia needs to be managed tactfully to reduce morbidity and mortality, minimize disease transmission, protect health care personnel, and preserve health care system functioning in order to address patients with other problems which calls for preparedness of various departments. It is necessary to take appropriate infection control measures before imaging the next patient and to protect the radiology workforce to enable them to provide adequate services throughout the crisis.

Imaging does not have a significant role in screening or mild cases. HRCT has a role in the management of COVID patients with moderate or severe and/or worsening symptoms to look for complications. Differentials need to be considered while making the imaging diagnosis. Reporting templates should be adopted by radiology departments for prompt and uniform reporting.

## Conflict of Interest:

**None.**
